# Health Promoting Lifestyle Behaviors and Sleep Quality Among Saudi Postmenopausal Women

**DOI:** 10.3389/fpubh.2022.859819

**Published:** 2022-06-15

**Authors:** Enas Mahrous Abdelaziz, Nadia Bassuoni Elsharkawy, Sayeda Mohamed Mohamed

**Affiliations:** ^1^Department of Nursing, College of Applied Medical Sciences, Jouf University, Sakaka, Saudi Arabia; ^2^Department of Psychiatric Mental Health Nursing, Faculty of Nursing, Cairo University, Cairo, Egypt; ^3^Department of Maternal and Newborn Health Nursing, Faculty of Nursing, Cairo University, Cairo, Egypt

**Keywords:** sleep quality, promoting behaviors, menopause, women, lifestyle

## Abstract

**Background:**

One of the most effective measures regarding improving women's health is to promote healthy lifestyle behaviors. This study aimed to investigate the relationship between health-promoting behaviors and sleep quality among a sample of Saudi menopausal women.

**Methods:**

A descriptive cross-sectional study was used to collect data from 410 Saudi postmenopausal women visiting primary healthcare care centers located in Sakaka, Jouf, Saudi Arabia, using the Menopause Rating Scale (MRS), Health-Promoting Lifestyle Profile II (HPLPII) and Pittsburgh Sleep Quality Index (PSQI).

**Results:**

The mean age of the study participants was 52.60 ± 4.65 years, the study findings highlighted that among all the HPLP domains, the highest mean score was observed for spiritual growth (24.00 ± 6.60) whereas the lowest score was observed for physical activity (16.18 ± 1.8). Statistically significant negative relations between the total score of HPLP and sleep quality score and menopausal symptoms (*p* < 0.001, *p* < 0.005), respectively. Total scores of lifestyle, health responsibility, and stress management were significant differences between participants with good and poor sleep quality. Being overweight/obese, physically inactive, having a chronic illness and poor sleep quality were predictors influencing health-promoting behavior.

**Conclusion:**

Health-Promoting Lifestyle Profile II was more obvious in the good sleeper in form of health responsibility and stress management. Being overweight and or obese, having a chronic illness, and having poor sleep quality were the significant factors influencing health-promoting behaviors. Designing and performing educational interventional plans are crucial to create motivation toward a healthy lifestyle and improve the quality of their sleep.

## Introduction

Menopause is the permanent cessation of menstruation for at least 1 year after the last menstruation without other reasons, such as chemotherapy, gland disorders, and removal of the uterus (1). Menopause is characterized by a wide range of psychological and physiological symptoms, including urogenital manifestations (sexual problems and urinary incontinence), somatic manifestations (heart discomfort, hot flashes, and sleeping difficulties), and psychological manifestations (distress, mood swings, and anxiety), all of these symptoms influence women's general wellbeing and reducing the quality of life (2, 3). The prevalence of postmenopausal symptoms can be influenced by socio-demographic, psychosocial, cultural, and lifestyle factors, which differ markedly among women all over the world (4).

The exact cause of sleep disorders in postmenopausal women is unknown, and it seems to vary based on the particular symptoms of sleep disorders. Menopause, aging, vasomotor symptoms, distress, anxiety, and many other health conditions, including cardiovascular disease, endocrine disease, medication, and psychosocial factors, are all potential factors of sleep disorders (5). One of the most common reasons for referring postmenopausal women to health care facilities and using tranquilizers is sleep disorders. The prevalence of sleep disorders ranges from 28 to 63%, and approximately one-third of postmenopausal women suffer from sleep disturbances (6, 7), including difficulty falling asleep and or maintaining sleep resulting in somnolence and fatigue during the day (8).

Health promotion is an essential health aspect, and its purpose is to enable people to gain, maintain or improve their control over their health. Health promotion is also defined as “a basic health management strategy that means adopting patterns that support health to change behavior and increase people's quality of life (9).” Health-promoting lifestyle (HPL) is essential for empowering individuals to achieve optimal health, prevent illness, and healthy lifestyle (10). Healthy lifestyle behaviors including regular physical activity, proper nutrition, and health responsibility; stress management, interpersonal relationships, and spiritual growth are all examples of health-promoting behaviors (7, 11). HPL has obvious benefits in many aspects of postmenopausal women's life, leading to physical and emotional changes, sleep, and cardiovascular health. Unhealthy lifestyle, including lack of physical activity, poor eating habits, and insufficient stress relief, exacerbates the consequences of postmenopausal women's hormonal changes and lowers the quality of life (12).

Concerns regarding health-promoting behavior and sleep problems are increased as the population of postmenopausal women rises. Furthermore, only a few studies have researched health-promoting lifestyle behavior and sleep quality in Saudi postmenopausal women and, there is limited literature available from the Arab countries. The present study will add to the literature on postmenopausal HPL behavior and their associations with sleep quality in Saudi women. The present study objective was to assess the relationship between health-promoting lifestyle behaviors and sleep quality in Saudi postmenopausal women.

## Materials and Methods

### Design, Setting, and Participants

A descriptive cross-sectional study was conducted from December 2020 to May 2021. The present study was the second phase of the project “Sleep problems and health-promoting behaviors in menopausal women,” which was registered under number 1384754968. Among the 23 primary healthcare centers (PHCC) in Sakaka-Jouf, Saudi Arabia, 5 PHCC were randomly selected for data collection by simple random sampling technique. Postmenopausal women who attend or accompany other patients to the chosen PHCC were asked to participate in the study. The questionnaires were distributed by the researchers to all participants using face-to-face interviews, except those women who refused to participate. The inclusion criteria were menopausal women between 50 and 60 years, had experienced amenorrhea for at least 1 year (12 consecutive months without menstruation). All women undergoing hysterectomy, on hormonal replacement therapy (HRT), or who had a history of using the psychotropic medication, had cognitive impairments, or had physical handicaps were excluded from this study.

The sample size was established based on the total population size of 12,634 women aged 50–60 years in Sakaka, Jouf, Saudi Arabia using Epi- Info version 7 statistical sample size calculators. A convenience sample of 373 was determined with an estimated response rate of 50%, a confidence interval of 95%, and a margin of error of 5%. After accounting for 10% attrition or incomplete responses, the sample size was increased to 410 postmenopausal women.

### Measures

The study utilized a four-structured sectioned questionnaire based on the study objectives after extensive literature reviews of similar research articles.

#### Sociodemographic Data

It consisted of data related to age, marital status, education, occupation, and presence of chronic illness, age at menopause onset, menopause duration, and parity. This section also assessed the participants' physical activity, and body mass index (BMI), Physical activity was identified as any exercise for 20–30 min in the form of walking or domestic activities. Physical activity was classified into three categories: infrequent or less three times a week, average or from three to five times a week, and frequent or more five times a week. Furthermore, participants' height and weight were measured to calculate their BMI using the formula weight/height^2^ and reported in kilograms per square meter.

#### Menopausal Rating Scale (MRS)

It was developed by Heinemann et al. (13) to assess the frequency and severity of menopausal symptoms. It consists of 11 items (symptoms or complaints). It categorized under three subscales: psychological symptoms (four items; disturbances of women's psychological states such as depressive mood, nervousness, anxiety, and other disturbances in the psychological state of women), and symptoms from Soma (four items), (Hot flushes/sweating, heart discomfort, sleep problems, and joint and muscular discomfort), and urogenital symptoms (three items; disturbances of women's urinary and sexual status such as sexual problems, bladder problems, and dryness of vagina). Each item in the scale is scored on a 5 point scale starting from 0 indicating (no symptoms) to 4 indicating (very severe symptoms). Total scoring is obtained by adding all the points of each item and ranges between 0, (asymptomatic), and 44, which indicates the highest degree of complaint. The current study employed an Arabic version of the MRS, which had previously been validated in an Egyptian study by Sweed et al. (14). Summation scores for severity were classified as follows: none/little = 0–4, mild = 5–8, moderate = 9–16, severe/very severe = 17+.

#### Health Promoting Lifestyle Profile II (HPLP II)

Health Promoting Lifestyle Profile II was developed by Walker et al. (15) to assess health-promoting behaviors. It consists of 52 items classified into six domains; physical activity (eight items), health responsibility (nine items), nutrition (nine items), interpersonal relationships (nine items), spiritual growth (nine items), and stress management (eight items). The total score ranges between 52 and 208. Items were scored on a four-point Likert scale for each item ranging from 1 (never) to 4 (routinely). The HPLP II scores are divided into four levels: poor (range 52–90), moderate (range 91–129), good (range 130–168), and excellent (range 169–208). High scores on all subscales indicate better adherence to health-promoting behavior. Cronbach's alpha for the original version of the HPLP II was 0.94, and it ranged from 0.79 to 0.87 for the six subscales. In this study, the Arabic version of the tool was used, and the Cronbach's alpha coefficient of internal consistency of total HPLP was = 0.89 (16).

#### Pittsburgh Sleep Quality Index (PSQI)

It is an efficient tool for measuring the subjective quality of sleep and sleep patterns. It was developed by Buysse et al. (17) and used in Arabic culture (18). It distinguishes “poor” from “excellent” sleep by evaluating seven domains; subjective sleep quality, sleep latency, duration, efficiency, and disturbances, use of sleeping medications, and daytime dysfunction. A total sum of 5 or higher defines a “poor” sleeper. The scale had good internal reliability (Cronbach's Alpha value = 0.872). A 10% pilot study was carried out to test the clarity of the questionnaire. The pilot study's data was not included in the current study.

### Ethical Considerations

Ethical clearance was obtained from the Local Committee of Bioethics (LCBE), the institutional review board of Jouf University (No: 03-03/42), and the data collection was started after ethical approval. All procedures were done based on the declaration of Helsinki. The participants were informed of the objectives and expected outcomes of the study. They were informed that they had the right to withdraw from the study at any time without giving reasons. Informed consent was obtained from all eligible participants who agreed to participate in the study. The confidentiality of data and the privacy of women were protected. Researchers wrote and kept a code number to keep postmenopausal women anonymous.

### Statistical Analysis

Data were fed into the computer and analyzed using IBM SPSS software package version 24.0 (IBM Corp., Armonk, NY, USA). Qualitative data are described using numbers and percentages and quantitative data were described using mean and standard deviation. The significance of the obtained results was determined at the 5% level. The Pearson correlation coefficient was used to determine the relationship between the variables. The student *t*-test was used to compare between two groups. Multiple linear regression was performed to identify the most independent factor influencing health-promoting behavior.

## Results

**Figure 1:**
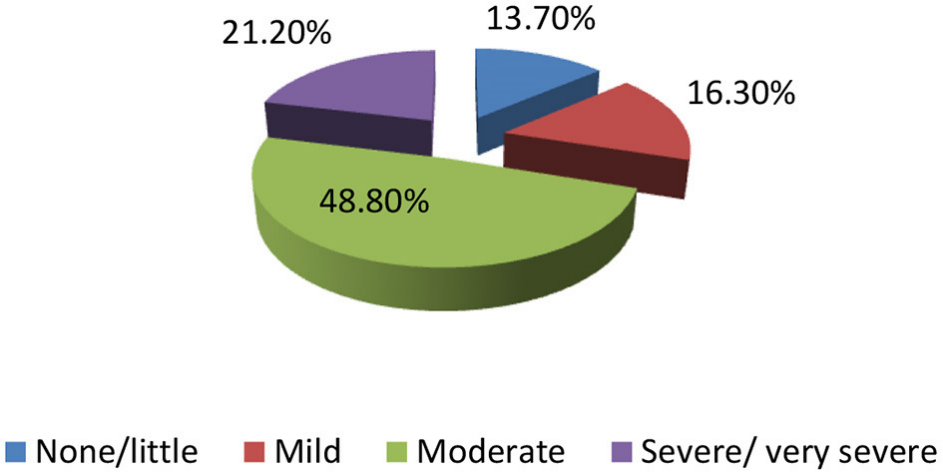
Severity of the menopausal symptoms among postmenopausal women (*n* = 410).

Table 1 illustrates sociodemographic characteristics of 410 postmenopause women with a mean age of 52.60 ± 4.65 years, their age of menopause was 50–55 years among 61.2%, and menopause duration was <5 years in 46.6% of the participants. More than two-thirds of women were educated (66.8%) (Various educational levels (from intermediate to university and above), 77.8% were married, 64.4% being housewives, and 66.3% having a chronic illness. More than three-quarters (78.8%) of the participants were physically inactive and BMI varied between overweight and obese (73.7%). About half of the participants (48.8%) had moderate levels of menopausal symptoms, and 21.20% had severe symptoms (Figure 1).

**Table 1:** Socio-demographic characteristics of the participants.

**Characteristics**	**Total (*****n*** **= 410)**	
	**No**.	**%**
**Age (years)**		
50–52	189	46.1
53–55	147	35.9
56–60	74	18.0
Mean ± SD	52.60 ± 4.65	
**Age at menopause**		
<50 years	88	21.5
50–55 years	251	61.2
≥55 years	71	17.3
Mean ± SD	51.14 ± 3.06	
**Menopause duration**		
<5 years	191	46.6
5–10 years	173	42.2
10 years and more	46	11.2
Mean ± SD	4.50 ± 3.84	
**Education**		
Cannot read and write/primary	136	33.2
Intermediate/secondary	202	49.3
University/Master-PhD	72	17.5
**BMI (kg/m** ^**2**^ **)**		
Underweight <18.5	22	5.3
Normal–Health weight 18.5–24.9	86	21.0
Overweight 25–29.9	139	33.9
Obese (≥30.0)	163	39.8
Mean ± SD	28.07 ± 6.72	
**Parity**		
Nulliparous	69	16.8
Parous	341	83.2
Mean ± SD	5.11 ± 1.62	
**Marital status**		
Single	12	2.9
Married	319	77.8
Widowed and or divorced	79	19.3
**Occupation**		
Housewife	264	64.4
Employee	106	25.8
Retired	40	9.8
**Physical activity (times/week)**		
<3 times	323	78.8
3–5 times	72	17.6
>5 times	15	3.6
**Having chronic illnesses**		
Yes	272	66.3
No	138	33.7

The overall menopausal symptoms score was (11.77 ± 6.75) indicating a moderate level of menopausal symptoms severity. The highest score was in somatic symptoms followed by urogenital symptoms, and the least one is psychological (4.26 ± 2.59, 3.90 ± 2.74, and 3.61 ± 3.47, respectively; Table 2). The total mean score HPLP-II was (124.72 ± 16.05) indicating a moderate level of HPLP-II with the highest mean score of spiritual growth (24.00 ± 6.60) and the lowest mean score of physical activity (16.18 ± 1.8; Table 3).

**Table 2:** Descriptive analysis of the studied women according to menopausal symptoms (*n* = 410).

**Menopausal symptoms**	**Total score**	**Percent score**
	**Mean ± SD**	**Mean ± SD**
Somatic symptoms	4.26 ± 2.59	35.49 ± 21.56
Urogenital symptoms	3.90 ± 2.74	24.38 ± 17.15
Psychological symptoms	3.61 ± 3.47	22.55 ± 21.67
Overall menopausal symptoms	11.77 ± 6.75	27.74 ± 15.35

**Table 3:** Descriptive analysis of the studied women according to health promoting behavior (*n* = 410).

**Health promoting behavior**	**Percent score**	**Range**
	**Mean ± SD**	
Interpersonal relations	19.75 ± 6.33	1–9
Spiritual growth	24.00 ± 6.60	1–9
Health responsibility	21.15 ± 5.40	1–9
Stress management	22.51 ± 4.33	1–8
Nutrition	21.13 ± 6.22	1–9
Physical activity	16.18 ± 1.8	1–8
Total score of lifestyle	124.72 ± 16.05	1–9

Pearson correlation coefficient test showed a significant negative correlation between total sleep quality scores and HPLP-II in terms of health responsibility, physical activity, and stress management (*p* < 0.001, 0.001, and 0.013). Low physical activity and poor stress management increased somatic symptoms (*p* < 0.001, *p* = 0.037). Psychological symptoms was negatively correlated with spiritual growth, stress management, and total HPLP score (*p* = 0.024, *p* = 0.010, *p* < 0.001). Poor HPLP in terms physical activity potentiate urogenital symptoms (*p* = 0.028, *p* = 0.047). Total MRS had a significant negative correlation with health responsibility, physical activity, and total score of HPLP (*p* = 0.048, *p* = 0.047, *p* = 0.005; Table 4).

**Table 4:** Correlation between HPLP-II, sleep quality, and the menopausal symptoms.

**Variables**		**HPLP-II**						
		**Health responsibility**	**Physical activity**	**Nutrition**	**Spiritual growth**	**Interpersonal relations**	**Stress management**	**Total score of HPLP**
Total sleep quality scores	*r*	−0.205^*^	−0.159^*^	0.142	−0.091	0.094	−0.122^*^	−0.260^*^
	*p*	<0.001^*^	0.001^*^	0.208	0.064	0.065	0.013^*^	<0.001^*^
Somatic symptoms	*r*	−0.094	−0.163^*^	−0.067	−0.097	−0.084	−0.189^*^	−0.088
	*p*	0.057	0.001^*^	0.173	0.491	0.360	0.037	0.075
Psychological symptoms	*r*	0.021	−0.056	0.028	−0.052^*^	0.015	−0.127^*^	−0.238^*^
	*p*	0.673	0.259	0.574	0.024	0.759	0.010^*^	<0.001^*^
Urogenital symptoms	*r*	0.001	−0.213^*^	0.037	−0.093	−0.031	−0.029	−0.194^*^
	*p*	0.987	0.028	0.632	0.073	0.536	0.561	0.047
Total MRS	*r*	−0.120^*^	−0.098	0.052	−0.086	−0.019	−0.056	−0.140^*^
	*p*	0.048	0.047	0.297	0.081	0.697	0.255	0.005^*^

*r, Pearson coefficient; HPLP-II, Health Promoting Lifestyle profile-II*.

* *Statistically significant at p ≤ 0.05*.

*DF = 408*.

In classifying good vs. poor sleep, 228 (55.6%) participants reported good sleep quality and had global scores ≤5, whereas 182 (44.4%) reported poor sleep quality (PSQI score >5). The total mean score of HPLP-II was higher in participants with good sleep quality and lower with poor sleep (132.08 ± 17.25 vs. 117.3 ± 18.88, *p* < 0.001). The score for health responsibility and stress management dimensions of HPLP was significantly higher in women with good sleep quality (*p* < 0.001; Table 5).

**Table 5:** The mean scores of HPLP-II in menopausal women with good and poor sleep quality.

**HPLP-II**	**Sleep quality ± SD**		***t*** **-value**	***p*** **-value**
	**Poor >5 (*n* = 182) 44.4%**	**Good ≤5 (*n* = 228) 55.6%**		
Health responsibility	18.10 ± 5.20	24.20 ± 5.59	3.713^*^	<0.001^*^
Physical activity	16.12 ± 1.91	16.23 ± 1.69	0.586	0.558
Nutrition	20.54 ± 5.97	21.72 ± 6.47	1.802	0.072
Spiritual growth	23.26 ± 6.60	24.70 ± 6.56	0.640	0.522
Interpersonal relations	19.10 ± 6.01	20.40 ± 6.65	1.942	0.053
Stress management	20.18 ± 4.70	24.83 ± 3.96	2.495	<0.001^*^
Total score of lifestyle	117.3 ± 18.88	132.08 ± 17.25	3.822^*^	<0.001^*^

*T, student t-test; SD, standard deviation; HPLP-II, Health Promoting Lifestyle profile-II*.

*DF = 408*.

* *Statistically significant at p ≤ 0.05*.

Multiple linear regression analysis was conducted to identify the factors influencing HPLP-II. The results showed that the four factors that negatively affect HPLP-II are as follows; being obese (*p* = 0.026), physically inactive (*p* = 0.037), having a chronic illness (*p* = 0.014), and poor sleep quality (*p* = 0.048; Table 6).

**Table 6:** Multiple linear regression analysis of the factors influencing heath promoting behavior.

**Variable**	**B (95% CI) coefficient**	**SE**	**Beta**	***t*** **-value**	***p*** **-value**	**95% CI**	
						**LL**	**UL**
Body Mass Index (BMI)	−0.623	0.280	−0.112	2.228^*^	0.026^*^	−1.173	−0.073
Education	−0.046	0.235	−0.010	0.195	0.846	−0.507	0.416
Having chronic illness	−1.528	0.620	−0.124	2.462^*^	0.014^*^	−2.747	−0.308
Physical activity	−0.364	0.086	−0.253	4.573^*^	0.037^*^	−0.212	0.526
Quality of sleep	−0.146	0.074	−0.101	1.987^*^	0.048^*^	−0.291	−0.002

R^2^, 0.018; F, 3.186^*^; p = 0.008^*^.

*f and p, f and p values for the model; R^2^, coefficient of determination; B, unstandardized coefficients; SE, estimates standard error; Beta, standardized coefficients; t, t-test of significance; CI, confidence interval; UL, upper limit; LL, lower limit*.

* *Statistically significant at p ≤ 0.05*.

## Discussion

This study was carried out for the purpose of determining the relationship between health-promoting behaviors, and sleep quality among a sample of Saudi postmenopausal women aged 50–60 years. It was clear that in this study, postmenopause women had reported moderate severity (9–16) of symptoms of the menopausal rating scale (MRS), the total mean MRS score was in the same line of new Saudi study result (4), while other Saudi study reported mild symptoms (19, 20).

Du et al. (21) found the role of such participants' characteristics as socioeconomics factors, lifestyle, and education in explanation of study results differences; moreover, individual variability responses to menopause and estrogen deficiency. Huang et al. (22) concluded that women who had moderate to severe symptoms were more likely to visit the hospital and increase the burden on the healthcare system.

Regarding subscales of menopause rating scales, the first rank was somatic symptoms, followed by urogenital symptoms and the last one was psychological symptoms. The increased frequency of somatic symptoms may be due to hormonal depletion that can increase symptom complaints during this period and cause such irritant symptoms (precisely hot flushes and night sweating). Moreover, higher somatic symptoms could explain as lacking participants' awareness of the availability of therapies such as hormonal replacement therapy and psychological therapies that could minimize/alleviate the severity of symptoms. This result is in the same line as other studies (4, 20, 23–25), as the intensity of symptoms may fluctuate depending on how these symptoms are perceived in different nations. Therefore, the appearance of these symptoms should be considered by professionals. The current study manifested urogenital symptoms were a second rank after somatic symptoms. This result could explain as most the Saudi women didn't report urogenital and sexual problems/dysfunction to health care providers due to social barriers or they may believe that those symptoms are a natural part of aging. This result is in the same line with a recent study (26); another Spanish study indicated a higher prevalence of genitourinary symptoms up to 70% of menopausal women (27).

Improving a healthy lifestyle is an effective way to manage menopausal symptoms, and eventually lower healthcare costs (12). Moreover, complications associated with menopause can be minimized if women adopt health-promoting behaviors such as self-actualization, self-responsibility, exercise, nutrition, stress management, and interpersonal relationship. The present study revealed that the studied menopausal women had moderate health-promoting behavior as the participants' health-promoting lifestyle mean score was 124.72 ± 16.05, ranging from 91 to 129. This result may be may be to sociodemographic factors lifestyle, and availability of health services. The present study matched the findings of another study by Ref. (7, 23, 28, 29). On the contrary, the overall score for health-promoting lifestyle practices was at a low level as a recent Pakistan study (3). This is most likely due to cultural disparities in health behaviors among women from other nations. Policymakers and healthcare providers must pay more attention to promoting a healthy lifestyle, sleep quality, and overall quality of life.

Regarding health-promoting behavior subscales, it was observed that the highest scores were in spiritual health, one of the most essential aspects of human health as it provides people with meaning and direction in life, as well as traits such as life stability, peace, and a sense of closeness to oneself, God, and others (30, 31). The current result could interpret in form of the important rules of the Islamic religion and spiritual enhancement in Saudi Arabia. This result is in harmony with (29, 32), and another Turkish study (9). Stress-related health problems are common among middle-aged women. The present study showed that stress management scores were high among study participants. This may interpret as Saudi postmenopause women are closely considered Islamic culture and values that encompassed new roles of menopause women as mentoring younger generations and instilling strong cultural teachings, so they may be less stressed and capable to overcome this stage. This finding is consistent with a study of Iranian women (33). Biological responses to stress may play a role in the development of physiological hazards. Thus, nurses should educate menopause women about sources of stress and its causes as well as strategies to reduce stress.

Moreover, our study participants' health responsibility scores were considered high, this could interpret in form of increased participants' awareness of their health responsibilities, and availability of free health services in Saudi Arabia that could improve health control among participants (34), this finding contradicts the findings of other studies by Malik et al. (3) and Alotaibi et al. (29).

Generally, women attain menopause unprepared to deal with the changes that come with this stage of life, in addition to insufficient knowledge of well-balanced diet, may lead to lack of essential nutrients, obesity, and diseases, current findings indicated nutrition score was not high. This result may encompass certain as being overweight and or obese and having more than five children, thus their awareness of a healthy well-balanced diet was inadequate, which is similar to the result of the (4, 35). According to world Health Organization (WHO) recommendations regarding nutrition, menopausal women's physiological nutrition results in a reduction in morbidity and unfavorable symptoms of menopause.

The current findings indicated that about more than three- quarter (78.8%) of women were physically inactive. These findings were clear in Arab communities, as the physical activity did not perceive as part of a healthy lifestyle, also women may express a lack of positive attitude toward physical activity because of cultural or personal reasons. This result is on the same track as similar study results (3, 4, 9, 29).

Findings showed a significant negative relationship between total scores of both health-promoting behavior and both menopausal symptoms and sleep quality. These findings were in the same line with (23, 36, 37). Based on these results, the absence or negative of healthy life habits can influence the dimension of self-care/search for health care at this stage and increase the incidence and intensity of menopausal symptoms and worsen sleep quality. Frange et al. (38) added that, poor sleep quality raises the chance of women's quality of life deteriorating by six–eight times. In the same direction, Malik et al. (1) showed the effect of lifestyle modification programs on promoting all dimensions of life quality and reduction of menopausal symptoms. Accordingly, Silva et al. (39) mentioned that participants with poor sleep quality didn't exacerbate symptoms of menopause only, but also trigger factors that potentiate physical hazards. Therefore, designing and implementing intervention programs are highly essential aiming to moderate menopausal symptoms and improve sleep quality. Currently, no evidence for the relation between total HPLP scores and somatic symptoms subscale of the Menopause Rating Scale (MRS). This could interpret as participants' perception of somatic symptoms as a normal-aged manifestation.

Physical activities are important in all age groups, particularly in middle-aged women, and insufficient physical exercise causes physical hazards. The present study showed a negative correlation between physical activity and sleep quality as decreasing physical activity, increasing sleep quality score (women with higher sleep quality score considers poor sleeper), 73.7% of women were overweight to obese in this study. This may be related to a lack of physical activity, being housewife. Poor sleep quality leads to a sedentary lifestyle that lead to alteration of physical exercise and potentiation of hazardous of overweight and obesity (40). This result is supported by other studies (7) who reached to the conclusion that poor sleep quality was linked to a decrease in the level of physical activity, as farm women were physically active than housewives.

In this study, about half (44.4%) of participants have poor sleep quality. These findings may be as a result of physiological and psychological changes prior and during menopause that induce sleep problems. This percent deserves attention as they are not diagnosed, nor treated in health services. Furthermore, this percentage isn't only affecting women's daily functions but in the long run, it also raises the risk of cardiovascular disease and depression. The current findings in the same line with (7), while in the opposite side of Chinese study (36, 40, 41). This difference in studies results may relate to variability of assessment methods of sleep quality. A statistically significant differences between total mean scores of health promoting behaviors among poor and good sleeper were reported. Women with good sleep quality had a significantly higher total lifestyle score than women with poor sleep quality (132.08 ± 17.25 vs. 117.3 ± 18.88, *p* < 0.001). The scores for health responsibility and stress management were significantly higher in women with good sleep quality. Regarding stress management dimension, this may attribute to availability different sources of knowledge about menopausal symptoms and how to deal with it from relatives, friends, media, and personal experience. Saudi Arabian culture characterized by providing each other support in both good and stressful times, also could interpret the influence of strong relationship.

Predictors of having poor health promoting behaviors were being obese, physically inactive, having chronic illness, and poor sleep quality. It's obvious that overweight/obesity and its associated physical complications could interfere with daily functioning. History of chronic illnesses can be interpreted by the exaggerating effects of the chronic disease itself, psychological effects because of chronic disease, and the aging process that may all affect quality of life. This is consistent with Lee et al. (5). Previous literature clarified that, poor sleep quality during menopause occur in response to frequent awakening during the night that manifested in accordance with sweating symptoms from hot flashes.

Although literature supported the role of education on reporting good health-promoting behaviors through having proper access to health services and receiving better medical advice, as well as having a higher quality of life (3, 4, 25). The current findings didn't have evidence for the role of education regarding poor promoting behaviors, although Silva et al. (39) clarified the role of low education in negative perception of health. Also, it is unclear if someone with a higher education comprehends the significance of certain health-promoting habits and behaviors.

### Limitations

The present study has some limitations including the nature of the study design (cross-sectional) so we cannot infer the causal relationship between the variables. All data was gathered by a self-reported survey; there is a risk of information bias. Because this study was conducted in Sakaka, Jouf, Saudi Arabia, the sample may not represent the overall Saudi female population. The intervening effect of aging may influence women who have gone through postmenopause.

### Clinical Implications/Recommendations

During the postmenopausal stage, healthcare providers and experts must be able to respond effectively to a woman's needs and problems. They should provide guidance and design awareness programs that highlight adopting healthy and active lifestyles, such as weight loss, physical activity, good nutrition, and maintaining regular medical follow-up, to improve general wellbeing and reduce sleep problems. Prospective longitudinal larger sample studies at different geographic regions in Saudi Arabia are recommended to obtain more comprehensive data on health-promoting behaviors and sleep quality in postmenopause.

Clinical implications include designing awareness programs and counseling sessions for postmenopausal women aimed at empowering them to engage in health-promoting behaviors. Post-menopausal clinics should be developed to promote healthy living and improve sleep quality. Healthcare professionals including nurses, gynecologists, and psychologists must be involved in counseling postmenopausal women on their physiological and psychological changes, educating them on maintaining better nutrition and physical activity for improving quality of life, and providing OTC supplements to alleviate menopausal symptoms.

## Conclusions

The findings of this study revealed such aspects that deserve to be considered in developing public policies aimed at this target group. The study's findings concluded that HPLP-II in postmenopausal women was moderate for the whole sample. Spiritual growth and stress management received the highest average scores from women, while physical activity received the lowest average score. HPLP-II was more explicitly found in good sleep quality in form of health responsibility and stress management. Being overweight or obese, physically inactive, having a chronic illness, and poor sleep quality were the significant factors influencing poor health-promoting behaviors.

## Data Availability Statement

The original contributions presented in the study are included in the article/supplementary material, further inquiries can be directed to the corresponding author.

## Ethics Statement

The studies involving human participants were reviewed and approved by Local Committee of Bioethics (LCBE), the Institutional Review Board of Jouf University (No: 03-03/42). The patients/participants provided their written informed consent to participate in this study.

## Author Contributions

EA and NE: idea, design, editing, and data collection. NE and SM: preparing the questionnaire. EA and SM: analyses. All authors: writing draft and approval of final draft. All authors agree to be accountable for the content of the work.

## Funding

Deputyship for Research & Innovation, Ministry of Education in Saudi Arabia is funding this research work through the project number 1384754968.

## Conflict of Interest

The authors declare that the research was conducted in the absence of any commercial or financial relationships that could be construed as a potential conflict of interest.

## Publisher's Note

All claims expressed in this article are solely those of the authors and do not necessarily represent those of their affiliated organizations, or those of the publisher, the editors and the reviewers. Any product that may be evaluated in this article, or claim that may be made by its manufacturer, is not guaranteed or endorsed by the publisher.
